# Supplementary material for: Primary School Pupils: Unequal GMC Developmental Pathways in a Single School Year
Source: Children (Basel). 2022 Jun 28;9(7):964. doi: 10.3390/children9070964 (PMC9319405; doi:10.3390/children9070964)
Supplement: Supplementary file 1 [file children-09-00964-s001.zip › children-1787267-supplementary.pdf]

children MDPI

**Table S1.** Number of children, mean age (±standard deviation) and mean score (±standard deviation) at T0 and T1 per grade, gender, and proficiency level for the walking backwards test.

| | | Overall group | | | | 'Low scores' category | | | | 'Average scores' category | | | | 'High scores' category | | | |
|---|---|---|---|---|---|---|---|---|---|---|---|---|---|---|---|---|---|
| | Grade | N | Age | T0 | T1 | N | Age | T0 | T1 | N | Age | T0 | T1 | N | Age | T0 | T1 |
| *Total* | 3rd | 382 | 6.5±0.5 | 25±11 | 29±11 | 61 | 6.5±0.5 | 10±3 | 19±7 | 248 | 6.5±0.5 | 24±6 | 29±10 | 73 | 6.6±0.5 | 41±10 | 35±11 |
| | 4th | 422 | 7.6±0.5 | 29±11 | 34±13 | 65 | 7.6±0.6 | 13±4 | 23±10 | 283 | 7.6±0.5 | 29±7 | 33±12 | 74 | 7.7±0.5 | 46±6 | 45±10 |
| | 5th | 425 | 8.6±0.5 | 34±12 | 40±13 | 72 | 8.4±0.4 | 17±4 | 30±10 | 278 | 8.6±0.5 | 33±6 | 40±12 | 75 | 8.6±0.5 | 52±4 | 52±9 |
| | 6th | 440 | 9.6±0.5 | 39±13 | 45±13 | 74 | 9.6±0.5 | 20±5 | 32±11 | 291 | 9.6±0.4 | 39±7 | 45±11 | 75 | 9.5±0.5 | 57±7 | 58±10 |
| | 7th | 474 | 10.6±0.5 | 41±13 | 45±13 | 72 | 10.5±0.5 | 20±6 | 31±10 | 314 | 10.6±0.5 | 41±8 | 46±11 | 88 | 10.5±0.5 | 59±6 | 56±11 |
| | 8th | 451 | 11.6±0.5 | 44±13 | 48±13 | 78 | 11.7±0.6 | 24±6 | 33±11 | 293 | 11.6±0.5 | 45±7 | 49±11 | 80 | 11.5±0.4 | 62±5 | 60±9 |
| *Boys* | 3rd | 170 | 6.5±0.5 | 22±10 | 26±10 | 29 | 6.4±0.5 | 9±2 | 18±7 | 106 | 6.5±0.5 | 21±6 | 27±10 | 35 | 6.6±0.5 | 37±7 | 32±9 |
| | 4th | 232 | 7.6±0.4 | 27±11 | 31±12 | 36 | 7.6±0.6 | 11±2 | 22±9 | 156 | 7.6±0.4 | 26±6 | 30±10 | 40 | 7.7±0.4 | 43±5 | 44±9 |
| | 5th | 209 | 8.6±0.4 | 32±12 | 38±14 | 39 | 8.4±0.4 | 17±4 | 28±11 | 135 | 8.6±0.4 | 31±6 | 37±12 | 35 | 8.5±0.5 | 51±5 | 53±9 |
| | 6th | 211 | 9.6±0.5 | 35±12 | 42±12 | 37 | 9.6±0.7 | 18±4 | 29±10 | 138 | 9.6±0.4 | 35±7 | 42±11 | 33 | 9.6±0.6 | 52±5 | 54±9 |
| | 7th | 217 | 10.6±0.5 | 38±12 | 42±12 | 33 | 10.5±0.5 | 18±4 | 28±8 | 143 | 10.7±0.6 | 38±6 | 43±10 | 41 | 10.5±0.5 | 54±5 | 51±11 |
| | 8th | 208 | 11.6±0.5 | 41±13 | 45±14 | 37 | 11.7±0.6 | 21±4 | 30±9 | 132 | 11.6±0.5 | 42±7 | 46±11 | 39 | 11.5±0.4 | 59±5 | 57±10 |
| *Girls* | 3rd | 212 | 6.5±0.4 | 27±12 | 31±11 | 32 | 6.5±0.5 | 11±4 | 19±7 | 142 | 6.4±0.4 | 26±6 | 31±10 | 38 | 6.6±0.5 | 45±10 | 38±11 |
| | 4th | 190 | 7.6±0.5 | 32±12 | 36±13 | 29 | 7.6±0.6 | 14 ±5 | 24±11 | 127 | 7.6±0.5 | 31±6 | 37±12 | 34 | 7.7±0.6 | 49±5 | 45±12 |
| | 5th | 216 | 8.6±0.5 | 36±11 | 43±12 | 33 | 8.4±0.4 | 18±4 | 33±9 | 143 | 8.6±0.6 | 35±6 | 42±11 | 40 | 8.6±0.5 | 53±4 | 52±10 |
| | 6th | 229 | 9.6±0.4 | 42±13 | 48±13 | 37 | 9.5±0.4 | 22±5 | 34±12 | 153 | 9.5±0.4 | 42±7 | 48±11 | 39 | 9.5±0.5 | 61±5 | 61±9 |
| | 7th | 257 | 10.5±0.5 | 44±14 | 48±13 | 39 | 10.5±0.5 | 22±7 | 34±12 | 171 | 10.6±0.5 | 43±8 | 48±11 | 47 | 10.6±0.5 | 62±4 | 59±9 |
| | 8th | 243 | 11.6±0.5 | 47±13 | 50±12 | 41 | 11.6±0.5 | 27±5 | 37±11 | 161 | 11.7±0.6 | 47±7 | 51±10 | 41 | 11.5±0.4 | 65±4 | 63±7 |

**Table S2.** Number of children, mean age (±standard deviation) and mean score (±standard deviation) at T0 and T1 per grade, gender, and proficiency level for the moving sideways test.

| | | Overall group | | | | 'Low scores' category | | | | 'Average scores' category | | | | 'High scores' category | | | |
|---|---|---|---|---|---|---|---|---|---|---|---|---|---|---|---|---|---|
| | Grade | N | Age | T0 | T1 | N | Age | T0 | T1 | N | Age | T0 | T1 | N | Age | T0 | T1 |
| *Total* | 3rd | 382 | 6.5±0.5 | 31±6 | 31±8 | 61 | 6.4±0.4 | 21±2 | 26±7 | 239 | 6.5±0.5 | 31±3 | 30±7 | 82 | 6.6±0.5 | 39±3 | 37±7 |
| | 4th | 422 | 7.6±0.5 | 36±7 | 38±8 | 70 | 7.5±0.4 | 26±3 | 32±8 | 249 | 7.6±0.5 | 35±3 | 38±7 | 103 | 7.7±0.5 | 44±3 | 44±7 |
| | 5th | 425 | 8.6±0.5 | 39±7 | 43±8 | 71 | 8.6±0.4 | 27±3 | 35±8 | 270 | 8.6±0.5 | 39±4 | 43±6 | 84 | 8.6±0.4 | 49±3 | 49±6 |
| | 6th | 440 | 9.6±0.6 | 43±8 | 46±8 | 72 | 9.6±0.6 | 31±4 | 39±6 | 284 | 9.6±0.4 | 43±4 | 46±7 | 84 | 9.5±0.4 | 54±4 | 51±8 |
| | 7th | 474 | 10.6±0.5 | 45±8 | 48±8 | 77 | 10.5±0.5 | 33±4 | 41±7 | 298 | 10.6±0.5 | 45±4 | 48±7 | 99 | 10.6±0.5 | 55±3 | 55±8 |
| | 8th | 451 | 11.6±0.5 | 47±9 | 51±9 | 78 | 11.7±0.6 | 34±4 | 42±8 | 293 | 11.6±0.5 | 47±4 | 51±7 | 80 | 11.6±0.5 | 60±5 | 59±7 |
| *Boys* | 3rd | 170 | 6.5±0.5 | 31±6 | 32±9 | 28 | 6.5±0.4 | 21±3 | 26±8 | 109 | 6.5±0.5 | 31±3 | 31±7 | 33 | 6.6±0.5 | 40±2 | 39±7 |
| | 4th | 232 | 7.6±0.4 | 36±7 | 39±8 | 36 | 7.6±0.5 | 26±3 | 32±7 | 144 | 7.6±0.4 | 35±3 | 38±7 | 52 | 7.7±0.5 | 44±4 | 45±7 |
| | 5th | 209 | 8.6±0.4 | 39±8 | 43±9 | 36 | 8.6±0.4 | 27±4 | 33±8 | 128 | 8.6±0.5 | 39±3 | 43±6 | 45 | 8.6±0.4 | 49±3 | 51±5 |
| | 6th | 211 | 9.6±0.5 | 43±9 | 46±9 | 32 | 9.7±0.7 | 30±3 | 39±6 | 140 | 9.6±0.4 | 43±5 | 46±8 | 39 | 9.5±0.5 | 55±4 | 52±9 |
| | 7th | 217 | 10.6±0.5 | 45±8 | 49±8 | 35 | 10.6±0.5 | 32±4 | 41±8 | 139 | 10.6±0.5 | 45±4 | 49±7 | 43 | 10.6±0.5 | 55±3 | 55±7 |
| | 8th | 208 | 11.6±0.5 | 47±9 | 51±9 | 34 | 11.7±0.6 | 34±5 | 42±6 | 139 | 11.5±0.5 | 47±4 | 51±7 | 35 | 11.6±0.4 | 61±4 | 59±8 |
| *Girls* | 3rd | 212 | 6.5±0.4 | 31±6 | 31±8 | 33 | 6.4±0.4 | 21±2 | 27±7 | 130 | 6.5±0.5 | 30±3 | 30±7 | 49 | 6.6±0.5 | 39±3 | 36±7 |
| | 4th | 190 | 7.6±0.5 | 35±6 | 38±8 | 34 | 7.5±0.3 | 25±3 | 31±7 | 105 | 7.6±0.5 | 35±3 | 38±7 | 51 | 7.7±0.6 | 43±3 | 43±7 |
| | 5th | 216 | 8.6±0.5 | 39±7 | 43±7 | 35 | 8.5±0.5 | 27±3 | 37±6 | 142 | 8.6±0.6 | 39±4 | 43±7 | 39 | 8.6±0.5 | 49±3 | 47±6 |
| | 6th | 229 | 9.5±0.4 | 43±8 | 46±8 | 40 | 9.5±0.4 | 31±5 | 39±6 | 144 | 9.5±0.4 | 43±4 | 46±7 | 45 | 9.5±0.3 | 54±3 | 51±7 |
| | 7th | 257 | 10.5±0.5 | 45±8 | 48±8 | 42 | 10.5±0.5 | 33±3 | 40±7 | 159 | 10.6±0.5 | 45±4 | 48±6 | 56 | 10.5±0.4 | 56±4 | 55±8 |

| | | | | | | | | | | | | | | | | | |
|---|---|---|---|---|---|---|---|---|---|---|---|---|---|---|---|---|---|
| | 8th | 243 | 11.6±0.5 | 47±8 | 51±9 | 44 | 11.7±0.6 | 35±4 | 41±9 | 154 | 11.6±0.5 | 47±4 | 51±7 | 45 | 11.6±0.5 | 59±5 | 59±5 |

**Table S3.** Number of children, mean age (±standard deviation) and mean score (±standard deviation) at T0 and T1 per grade, gender, and proficiency level for the jumping sideways test.

| | | **Overall group** | | | | **'Low scores' category** | | | | **'Average scores' category** | | | | **'High scores' category** | | | |
|---|---|---|---|---|---|---|---|---|---|---|---|---|---|---|---|---|---|
| | **Grade** | **N** | **Age** | **T0** | **T1** | **N** | **Age** | **T0** | **T1** | **N** | **Age** | **T0** | **T1** | **N** | **Age** | **T0** | **T1** |
| *Total* | 3rd | 382 | 6.5±0.5 | 38±10 | 43±12 | 60 | 6.4±0.4 | 24±3 | 32±8 | 254 | 6.5±0.5 | 37±6 | 42±9 | 68 | 6.6±0.5 | 55±5 | 56±8 |
| | 4th | 422 | 7.6±0.5 | 46±12 | 51±12 | 71 | 7.5±0.5 | 30±5 | 40±10 | 277 | 7.6±0.5 | 46±6 | 50±10 | 74 | 7.7±0.5 | 64±6 | 65±9 |
| | 5th | 425 | 8.6±0.5 | 53±12 | 58±13 | 72 | 8.4±0.4 | 34±6 | 44±12 | 271 | 8.6±0.5 | 53±6 | 58±11 | 82 | 8.7±0.5 | 70±5 | 70±10 |
| | 6th | 440 | 9.6±0.6 | 60±13 | 67±14 | 68 | 9.6±0.6 | 39±5 | 50±12 | 292 | 9.5±0.4 | 59±7 | 67±11 | 80 | 9.6±0.5 | 78±6 | 80±11 |
| | 7th | 474 | 10.6±0.5 | 64±12 | 70±14 | 79 | 10.6±0.5 | 46±7 | 55±11 | 310 | 10.6±0.5 | 64±7 | 69±11 | 85 | 10.6±0.5 | 82±6 | 84±10 |
| | 8th | 451 | 11.6±0.5 | 71±13 | 75±14 | 83 | 11.7±0.6 | 53±8 | 62±13 | 289 | 11.6±0.5 | 71±6 | 75±11 | 79 | 11.6±0.5 | 89±6 | 89±12 |
| *Boys* | 3rd | 170 | 6.5±0.5 | 40±10 | 43±12 | 27 | 6.5±0.4 | 25±3 | 34±8 | 111 | 6.5±0.5 | 39±6 | 41±10 | 32 | 6.6±0.5 | 56±4 | 56±10 |
| | 4th | 232 | 7.6±0.4 | 46±12 | 51±13 | 36 | 7.6±0.5 | 29±4 | 41±10 | 156 | 7.6±0.5 | 46±6 | 50±11 | 40 | 7.6±0.3 | 65±7 | 65±10 |
| | 5th | 209 | 8.6±0.4 | 53±13 | 57±14 | 35 | 8.5±0.4 | 33±6 | 41±12 | 134 | 8.6±0.5 | 53±6 | 59±11 | 40 | 8.6±0.4 | 70±6 | 69±12 |
| | 6th | 211 | 9.6±0.5 | 60±13 | 67±13 | 33 | 9.6±0.6 | 40±5 | 52±11 | 139 | 9.6±0.5 | 60±7 | 67±11 | 39 | 9.6±0.5 | 78±6 | 80±8 |
| | 7th | 217 | 10.6±0.5 | 66±12 | 71±13 | 39 | 10.7±0.6 | 48±6 | 57±12 | 140 | 10.6±0.5 | 66±6 | 71±10 | 38 | 10.5±0.5 | 83±4 | 85±8 |
| | 8th | 208 | 11.6±0.5 | 71±13 | 76±14 | 39 | 11.8±0.7 | 52±9 | 63±12 | 132 | 11.5±0.4 | 72±7 | 77±11 | 37 | 11.5±0.4 | 90±5 | 90±13 |
| *Girls* | 3rd | 212 | 6.5±0.4 | 36±10 | 42±11 | 33 | 6.4±0.4 | 24±2 | 31±8 | 143 | 6.5±0.4 | 35±5 | 42±9 | 36 | 6.6±0.5 | 54±5 | 56±7 |
| | 4th | 190 | 7.6±0.5 | 46±11 | 51±12 | 35 | 7.4±0.3 | 30±6 | 38±9 | 121 | 7.6±0.5 | 46±6 | 51±9 | 34 | 7.8±0.6 | 63±5 | 65±7 |
| | 5th | 216 | 8.6±0.5 | 53±12 | 59±13 | 37 | 8.4±0.4 | 34±5 | 46±11 | 137 | 8.6±0.6 | 52±6 | 58±10 | 42 | 8.7±0.5 | 69±5 | 71±9 |
| | 6th | 229 | 9.5±0.4 | 59±13 | 66±14 | 35 | 9.6±0.5 | 39±6 | 49±12 | 153 | 9.5±0.4 | 59±6 | 66±11 | 41 | 9.6±0.5 | 79±6 | 80±13 |
| | 7th | 257 | 10.5±0.5 | 63±13 | 69±14 | 40 | 10.5±0.5 | 44±6 | 53±10 | 170 | 10.5±0.5 | 63±6 | 68±10 | 47 | 10.6±0.6 | 81±7 | 83±12 |
| | 8th | 243 | 11.6±0.5 | 71±12 | 74±14 | 44 | 11.6±0.6 | 53±7 | 60±13 | 157 | 11.7±0.5 | 71±5 | 74±11 | 42 | 11.6±0.5 | 88±6 | 88±11 |

**Table S4.** Number of children, mean age (±standard deviation) and mean score (±standard deviation) at T0 and T1 per grade, gender, and proficiency level for the eye hand coordination test.

| | | **Overall group** | | | | **'Low scores' category** | | | | **'Average scores' category** | | | | **'High scores' category** | | | |
|---|---|---|---|---|---|---|---|---|---|---|---|---|---|---|---|---|---|
| | Grade | N | Age | T0 | T1 | N | Age | T0 | T1 | N | Age | T0 | T1 | N | Age | T0 | T1 |
| *Total* | 5th | 425 | 8.6±0.5 | 10±10 | 22±14 | 82 | 8.4±0.4 | 0±1 | 10±9 | 263 | 8.5±0.5 | 9±6 | 22±13 | 80 | 8.7±0.5 | 26±6 | 33±12 |
| | 6th | 440 | 9.6±0.5 | 18±13 | 25±13 | 73 | 9.5±0.4 | 2±2 | 11±10 | 288 | 9.6±0.7 | 16±9 | 24±11 | 79 | 9.7±0.5 | 38±6 | 37±10 |
| | 7th | 474 | 10.6±0.5 | 24±14 | 30±14 | 74 | 10.5±0.5 | 4±3 | 16±11 | 301 | 10.5±0.5 | 22±9 | 30±12 | 99 | 10.7±0.5 | 43±7 | 44±11 |
| | 8th | 451 | 11.6±0.5 | 32±15 | 38±14 | 71 | 11.6±0.6 | 10±6 | 26±13 | 292 | 11.6±0.5 | 31±9 | 38±12 | 88 | 11.6±0.5 | 51±6 | 49±13 |
| *Boys* | 5th | 209 | 8.6±0.4 | 13±10 | 25±14 | 37 | 8.4±0.4 | 1±1 | 14±10 | 132 | 8.6±0.4 | 12±6 | 24±13 | 40 | 8.8±0.5 | 29±4 | 36±11 |
| | 6th | 211 | 9.6±0.5 | 22±13 | 28±12 | 37 | 9.5±0.4 | 4±2 | 14±10 | 133 | 9.6±0.5 | 21±8 | 29±9 | 41 | 9.8±0.6 | 40±5 | 39±12 |
| | 7th | 217 | 10.6±0.5 | 29±14 | 35±14 | 33 | 10.6±0.6 | 6±4 | 19±13 | 132 | 10.6±0.5 | 28±8 | 34±11 | 52 | 10.6±0.5 | 46±6 | 48±8 |
| | 8th | 208 | 11.6±0.5 | 36±14 | 42±14 | 33 | 11.6±0.5 | 14±6 | 28±12 | 135 | 11.6±0.5 | 36±7 | 42±12 | 40 | 11.5±0.4 | 54±5 | 54±11 |
| *Girls* | 5th | 216 | 8.6±0.5 | 8±9 | 19±14 | 45 | 8.5±0.5 | 0±0 | 8±7 | 131 | 8.5±0.6 | 6±4 | 19±13 | 40 | 8.7±0.5 | 23±5 | 30±14 |
| | 6th | 229 | 9.5±0.7 | 14±12 | 21±13 | 36 | 9.4±0.4 | 0±1 | 8±9 | 155 | 9.5±0.4 | 12±7 | 20±12 | 38 | 9.5±0.4 | 36±6 | 36±9 |
| | 7th | 257 | 10.5±0.5 | 19±13 | 27±14 | 41 | 10.5±0.6 | 2±1 | 13±9 | 169 | 10.5±0.4 | 18±8 | 26±11 | 47 | 10.8±0.5 | 40±7 | 39±12 |
| | 8th | 243 | 11.6±0.5 | 28±14 | 34±13 | 38 | 11.6±0.6 | 6±4 | 24±15 | 157 | 11.6±0.5 | 27±8 | 34±11 | 48 | 11.7±0.5 | 48±6 | 44±12 |

**Table S5.** Percentage of children with negative (NED), no (NOD), and positive (POD) development per grade, gender, and proficiency level.

| | | | *WB* | | | *MS* | | | *JS* | | | *EHC* | | |
|---|---|---|---|---|---|---|---|---|---|---|---|---|---|---|
| | | | **NED** | **NOD** | **POD** | **NED** | **NOD** | **POD** | **NED** | **NOD** | **POD** | **NED** | **NOD** | **POD** |

| | | | | | | | | | | | | | |
|---|---|---|---|---|---|---|---|---|---|---|---|---|---|
| Grade | 3rd | 24.1% | 18.1% | 57.9% | 33.2% | 28.0% | 38.7% | 10.2% | 47.9% | 41.9% | | | |
| | 4th | 28.2% | 16.4% | 55.5% | 18.2% | 29.6% | 52.1% | 13.0% | 41.2% | 45.7% | | | |
| | 5th | 21.2% | 14.6% | 64.2% | 17.2% | 23.5% | 59.3% | 15.3% | 34.6% | 50.1% | 9.2% | 15.8% | 75.1% |
| | 6th | 19.3% | 19.1% | 61.6% | 22.0% | 21.1% | 56.8% | 11.4% | 33.4% | 55.2% | 16.6% | 17.0% | 66.4% |
| | 7th | 24.5% | 16.5% | 59.1% | 20.5% | 23.4% | 56.1% | 10.5% | 42.0% | 47.5% | 15.6% | 18.6% | 65.8% |
| | 8th | 26.6% | 19.3% | 54.1% | 18.2% | 23.7% | 58.1% | 12.4% | 43.7% | 43.9% | 19.3% | 15.7% | 65.0% |
| Gender | Boys | 23.3% | 17.5% | 59.3% | 20.9% | 23.3% | 55.8% | 13.7% | 38.3% | 48.0% | 16.3% | 15.1% | 68.5% |
| | Girls | 24.6% | 17.1% | 58.2% | 21.7% | 26.2% | 52.1% | 10.7% | 42.3% | 47.0% | 14.3% | 18.3% | 67.4% |
| Proficiency level | Low scores | 6.6% | 11.1% | 82.2% | 9.1% | 16.1% | 74.8% | 5.3% | 32.1% | 62.6% | 0.7% | 20.7% | 78.7% |
| | Average scores | 22.3% | 18.1% | 59.6% | 19.4% | 24.6% | 56.0% | 10.9% | 40.3% | 48.8% | 13.1% | 14.9% | 72.0% |
| | High scores | 46.0% | 20.0% | 34.0% | 37.2% | 32.3% | 30.5% | 23.1% | 48.1% | 28.8% | 35.0% | 19.9% | 45.1% |

WB: walking backwards; MS: moving sideways;, JS: jumping sideways; EHC: eye-hand coordination.
